# Development of a nationwide telepathology consultation and quality control program for cancer diagnosis in China

**DOI:** 10.1186/1746-1596-8-S1-S6

**Published:** 2013-09-30

**Authors:** Zhongjiu Zhang, Guangming Gao, Chen Zhou

**Affiliations:** 1 Bureau of Health Policy and Regulation, Ministry of Health, Beijing, China; 2 Division of Quality of Healthcare, Bureau of Health Policy and Regulation, Ministry of Health, Beijing, China; 3 British Columbia Cancer Agency, University of British Columbia, Vancouver, Canada

## Background

China has the largest numbers of cancer patients worldwide but has limited pathology resources. According to a survey done by Chinese Pathologists Association, a large number of pathologists in clinical practice in China has only one year of formal or informal training. Only in recent year, formal pathology residency programs similar to those of Western countries are available in a couple of large medical schools, where limited numbers of well-trained and experienced pathologists are located. When having rare or complex cancer pathology cases, pathologists often have difficulty in making an accurate diagnosis. Mistakes in cancer diagnosis are not uncommon, which often leads to inadequate treatment of patients and medical legal problems. Thus, there is a great need for pathology consultation and quality control of cancer diagnosis in China.

In recent years, telepathology or digital pathology has been applied in many areas of pathology, including remote consultation and quality control [1]. Difficult pathology cases can be sent through internet or intranet to remote site for consultation [2]. Quality control can be performed on digitalized image or WSI by expert pathologists at remote sites [3]. The practice of telepathology can solve the problem of healthcare system with poor pathology resource, especially in developing countries [4]. In order to use pathology resource efficiently and to improve the quality of pathology diagnosis of cancer, the Ministry of Health of China developed and launched a nationwide telepathology consultation and quality control program for cancer diagnosis in China in 2011.

## Designs

### Internet platform

**Figure 1:**
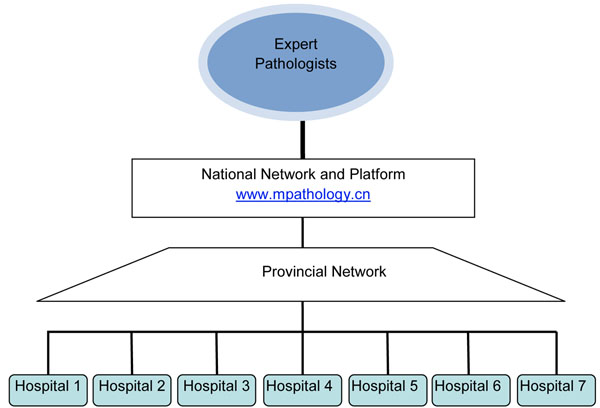
**Nation-wide Telepathology consultation and quality control network.** Figure 1 shows the flowchart of the internet platform and nation wide network. Participating hospitals send digital images or WSI of the histology slides of cancer through the internet to the platform, WSI is then stored in the server; expert pathologists from different institutions could access the website through the internet, review the WSI stored in the server and deliver a second opinion or make an assessment of quality of pathology diagnosis.

An internet based telepathology consultation platform (http://www.mpathology.cn) was designed to serve as the hub for the project, connecting hospitals and expert consultants. Selection an internet based platform or a website portal was due to the consideration that multiple hospitals/institutions and multiple expert pathologists from different institutions need to access the platform. A server was used for storage of images and data. Figure 1 shows the flowchart of the platform.

For telepathology consultation, referring pathologists from participating hospitals sent request for consults with attached WSI and related clinical information to the internet platform, the system would alert system information technologists by e-mails. The technologists would then check out the submitted materials to make sure the requesting form is filled out appropriately with the WSI attached. The system manager would then contact the expert pathologist for consultation.

For quality control, a designated expert pathologist would log into the platform, review the WSI of cancer case from specific hospitals, assesses the accuracy of the pathology diagnosis and quality of histology sections. The results would then be sent to provincial and national pathology quality control center.

All of the participating hospitals were supplied with a virtual microscope, Motic Virtual Microscopic Scanner (Motic Medical Diagnostic Systems Corporation, China). The virtual microscope and related software were tested and validated previously in a study involving a variety of 600 surgical pathology specimens in China. The study showed a high concordance rate (94.2%) between WSI and H/E glass slide [5].

### Expert consultants

356 pathologists with provincial and national reputation volunteered to participate in the program. 80 of them were selected to serve as expert consultants after passing an online assessment consists of 30 pathology consultation cases in WSI format. The names, affiliation of the pathologists and the areas of their subspecialty expertise were listed on the website (http://www.mpathology.cn). Experts were provided with a unique identifier and access code to the website. For telepathology consultation, the expert was instantly informed by the cell phone message and e-mail once he/she was requested for consult. The expert pathologist would use a computer or an Ipad to log into the website, check out the pending cases for consultation under his/her name, review WSI of the case, capture a representative image from WSI to included in the final report, type in pathologic features of the case, write up his/her opinion and provide a final diagnosis. After that, he/she would preview the final report, then sign the report with an electronic signature and then release the final report.

Once the final consultation report was released, the system sent an email to alert the system manager. The final consultation report was then sent by the system manger by e-mail or fax to the referring pathologist.

### Participating hospitals

87 hospitals in 17 provinces where national healthcare reform was carried out volunteered to participate the phase I of the program, all of the hospitals had more than 6000 pathology cases annually. Pathologists in those hospitals took an online diagnostic test of 50 cases of cancer. Those hospitals having pathologists with excellent test results were eliminated from the program. 60 hospitals were finally selected to participate in the phase I of the program. Participating hospitals were given a unique identification code and user name to login to the website platform. Pathologists of the participating hospitals are required to receive a short period of training, which includes operation of the virtual microscope and internet connection to the platform. Storage and security of the WSI, relevant clinical data, and consultation files are also to be addressed.

For telepathology consultation, each participating hospital is required to send WSI of at least 300 cases annually for second opinion. The number of cases is set at about 15% to 20% of cancer diagnosed each year in those hospitals. Hospitals can choose pathologists for second opinion or consultation from 80 expert consultants with different subspecialty expertise. For quality control of pathology diagnosis, the participating hospitals are required to submit WSI of every cancer diagnosed to the platform. Quality control is to be carried out by expert consultants every 3 month on 10% randomly selected cases. The accuracy of cancer diagnosis and quality of tissue section are to be reviewed, assessed, and submitted to provincial and national pathology quality control centers. The results of the phase I of the program are to be used to guide future deployment of the program across the country.

## Discussion and conclusion

Since 2009, due to the wide spread use of high speed internet and availability of low cost virtual microscopes in China, telepathology has gained attention from academic institutions and government. In 2010, the Ministry of Health of China released an announcement encouraging hospitals to use telepathology for cancer diagnosis and planned to develop a nationwide telepathology consultation service and quality control program. The plan is to set up in each province a provincial telepathology consultation and pathology quality control network, which also connects to a central national network. The goal of the plan is to facilitate remote consultation for cancer diagnosis and to monitor the accuracy of pathological diagnosis of cancer. In 2011, the phase I of the plan consisting of 60 hospitals in 17 provinces was completed.

In China, we do not know the exact number of error in pathological diagnosis of cancer, however, we believe the rate is much higher than those reported in Western countries, as many pathologists in China are not as well trained as those in Western countries and many pathologists in China do not have subspecialty expertise. The project we design asks participating hospitals to submit all of their newly diagnosed cancer cases. 10% of those cases will be randomly selected for review. The 10% is set arbitrarily and is not a high percentage for newly diagnosed cancer cases, in comparison to prospectively secondly review of all newly diagnosed cancer cases or retrospective mandatory review of all cancer cases by some institutions in North America.

Second opinion or consultation is extremely important in pathology diagnosis, especially in cancer diagnosis. In a retrospectively review of consultation cases of urological malignancy, Wayment *et al. *[6] reported that a 10% disagreement, of which 8% is major. Matasar *et al. *[7] review the difference between second opinion and submitting diagnosis in lymphoma cases and found that major diagnostic revision was 17.8% in 2001 and 16.4% in 2006. In Asia or developing countries, the discrepancy rate is similar or much higher. In a review of 673 consultation cases sent to a cancer center in Twain, Tsung [8] found a 16% of major disagreement between original diagnosis and second opinion. Hsu *et al*. [9] reported that among 2686 consultation cases, the tentative diagnosis and consultation diagnosis were discordant in 1,074 (64.3%) cases. Major discrepancy was seen in 205 (12.3%) cases, of which 66.8% were changed from malignant to benign, 21.0% were changed from benign to malignant. Based on these studies, the program we designed asks participating hospitals to submit 10-20% of their cases for consultation.

Through this program, we believe that in near future telepathology will be widely used in China for pathology consultation and quality control. The program will not only assists pathologists in dealing with diagnostically difficult pathology cases, but also benefits patients, who do not need to travel a long distance to large hospitals for a second opinion of cancer diagnosis, thus saving time and money for patients. The program will also guaranty the quality of pathology diagnosis of cancer in China.

## References

[B1] Rocha R Vassallo J Soares F Miller K Gobbi H Digital slides: present status of a tool for consultation, teaching, and quality control in pathology Pathol Res Pract 2009 205 11 735 41 10.1016/j.prp.2009.05.004 19501988

[B2] Wilbur DC Madi K Colvin RB Duncan LM Faquin WC Ferry JA Whole-slide imaging digital pathology as a platform for teleconsultation: a pilot study using paired subspecialist correlations 2010 19961250 10.1043/1543-2165-133.12.1949 PMC3694269

[B3] Ho J Parwani AV Jukic DM Yagi Y Anthony L Gilbertson JR Use of whole slide imaging in surgical pathology quality assurance: design and pilot validation studies Human pathology 2006 37 3 322 31 10.1016/j.humpath.2005.11.005 16613327

[B4] Hitchcock CL The Future of Telepathology for the Developing World Archives of pathology & laboratory medicine 2011 135 2 211 4 21284440 10.5858/135.2.211

[B5] Li X Gong E McNutt MA Liu J Li F Li T Assessment of diagnostic accuracy and feasibility of dynamic telepathology in China Human pathology 2008 39 2 236 42 10.1016/j.humpath.2007.06.008 17950781

[B6] Wayment RO Bourne A Kay P Tarter TH Second opinion pathology in tertiary care of patients with urologic malignancies Urol Oncol 2011 29 2 194 8 10.1016/j.urolonc.2009.03.025 19523859

[B7] Matasar MJ Shi W Silberstien J Lin O Busam KJ Teruya-Feldstein J Expert second-opinion pathology review of lymphoma in the era of the World Health Organization classification Ann Oncol 2011 21415238 10.1093/annonc/mdr029

[B8] Tsung JSH Institutional pathology consultation The American journal of surgical pathology 2004 28 3 399 10.1097/00000478-200403000-00015 15104305

[B9] Hsu CY Su IJ Lin MC Kuo TT Jung SM Ho DM Extra-departmental anatomic pathology expert consultation inTaiwan: a research grant supported 4-year experience J Surg Oncol 2010 101 5 430 5 20119977 10.1002/jso.21494

